# Developing the Nausea and Vomiting Thermometer Scale in children with cancer

**DOI:** 10.3906/sag-2005-88

**Published:** 2021-01-01

**Authors:** Aslı AKDENİZ KUDUBEŞ, Murat BEKTAŞ

**Affiliations:** 1 Department of Pediatric Nursing, Faculty of Health Sciences, Bilecik Şeyh Edebali University, Bilecik, Turkey; 2 Department of Pediatric Nursing, Faculty of Nursing, Dokuz Eylül University, İzmir, Turkey

**Keywords:** Nausea, vomiting, scale, children with cancer

## Abstract

**Background/aim:**

This study aimed to develop the Nausea and Vomiting Thermometer Scale (NVTS) in children with cancer.

**Materials and methods:**

This methodological study was conducted on 250 children with cancer at the research and training university hospital in Turkey between September 2019 and January 2020. The t-test, the ROC analysis, the Diagnostic index, and the Youden index were used for determining the scale of the cutting point. The regression analysis, the intra-class correlation coefficient, and the Bland–Altman analysis were used for the data analysis.

**Results:**

The scale-level content validity index was .94, which was coherent. As a result of the ROC analysis, the cut-off point was determined as three points. The NVTS showed good reliability, with an intra-class correlation coefficient of .99. In the linear regression analysis, a model was created for chemotherapy drugs, nausea and vomiting type, vomiting status, and the number of children with cancer who vomited explained 44.9% of their nausea and vomiting status. The results of the Bland–Altman analysis showed that the correlation coefficient between the differences and the means was insignificant.

**Conclusion:**

The NVTS was found to be a valid and reliable measurement tool for children with cancer in the Turkish sample.

## 1. Introduction

The new approaches used in the treatment of cancer improve children’s recovery rate. However, these methods can have various negative consequences on the child and the family. The use of high-dose drugs in chemotherapy causes children to experience many symptoms. Of these symptoms, fatigue, pain, oral mucositis, nausea, and vomiting are more common than the other symptoms [1–3].

Nausea and vomiting are common symptoms in children receiving cytotoxic chemotherapy. Many mechanisms are involved in the development of nausea and vomiting due to chemotherapy in children with cancer, damage to the blood-brain barrier, impaired gastrointestinal motility, and adrenal hormones [1,4]. The emetic effect of chemotherapy agents is more common in the first 24 h [5]. Uncontrolled nausea and vomiting cause physical effects, such as dehydration, electrolyte disturbance, malnutrition, gastrointestinal bleeding, and aspiration pneumonia in children [6,7].

The prevention of symptoms improves the quality of life of the child and contributes positively to treatment. Nurses working in the field of pediatric oncology have responsibilities for the evaluation, prevention, and alleviation of the nausea and vomiting experienced by children with cancer. Therefore, it is expected that evidence-based scientific knowledge obtained from research will be applied to nursing practice through monitoring the latest developments in this area. The first step in the prevention or alleviation of symptoms is a detailed diagnosis of the symptoms [4,8,9]. Therefore, the diagnosis of nausea and vomiting, which have serious effects on quality of life and treatment efficacy, is one of the most important factors [10].

Children cannot express symptoms such as pain, nausea, and vomiting, as adults do, due to language development and cognitive deficits. This situation makes the symptoms in children difficult to understand and complicated [11]. With the preschool period, children can use words and communicate to express the presence, intensity and absence of symptoms. Therefore, they can provide self-reporting, which is the most reliable method. However, cognitive development has not yet been completed in these children, as it continues until early adulthood. Therefore, communication between healthcare professionals and children is still limited due to language development or cognitive complexities. Therefore, the use of visual scales is recommended [12]. In addition, a scale should be accessible, easy to use, low cost and understandable in clinical settings [13]. Therefore, it is important to evaluate the symptoms with visual scales. Because visual scales both provide a clear demonstration of the symptom and are easy to apply, it prevents the loss of time and workforce for clinicians. In the literature, there is no cancer in children nausea and vomiting evaluating a visual scale in Turkey.

There are several national and international scales that evaluate nausea and vomiting in children with cancer [8]. Although there are studies for children to be assessed by medical staff for nausea and vomiting in Turkey, no scale has been developed specifically for children with cancer [14,15]. This limits the ability of nurses caring for children with cancer to diagnose nausea and vomiting symptoms and plan appropriate interventions. There is a need for a more reliable and valid tool for increasing the limited number of studies in Turkey.

## 2. Methods

### 2.1. Purpose

The aim of this study was to develop the Nausea and Vomiting Thermometer Scale (NVTS) in children who have been diagnosed with cancer for between 5 and 18 years in Turkey.

### 2.2. Sample Population

This study was conducted between September 2019 and January 2020 on children with cancer at the research and training university hospital in Turkey. The sample calculation was performed using the G*Power Software statistical analysis program 3.1. The scale developed in this study is not a Likert type scale, it is a visual analog type scale. For this reason, the sample calculation was made on the basis of regression analysis and ROC analysis. In the literature, it has been reported that the four variables that best determine nausea and vomiting in children with cancer are the type of chemotherapy drug, the presence, type and number of nausea and vomiting [4,5,15]. Considering the four variables to be used in the predictive validity of the procedure based on regression analysis, the effect size was calculated as 0.15 (medium), 80% power, and a 0.05 significance level, and the required sample size was 103 people. Considering a 10% loss, the sample size was planned to be 115 children. For this reason, the sampling involved 250 children aged between five and eighteen who volunteered to participate in the study. In this study, data were collected using the random sampling method.

The research inclusion criteria: Children aged between 5 and 18 who were receiving chemotherapy in the Pediatric Hematology-Oncology Clinic and Day Treatment Unit in Turkey and who volunteered to participate in the study. The research exclusion criteria were as follows: children who did not volunteer to participate in the study and were unconscious were not included.

### 2.3. Data Collection Tools

The child Information Form consists of seven questions used to obtain descriptive data about the children, such as their age, sex, diagnosis, chemotherapy drug, chemotherapy day, nausea and vomiting type, and vomiting status.

#### 2.3.1. Nausea and Vomiting Thermometer Scale

**Figure 1:**
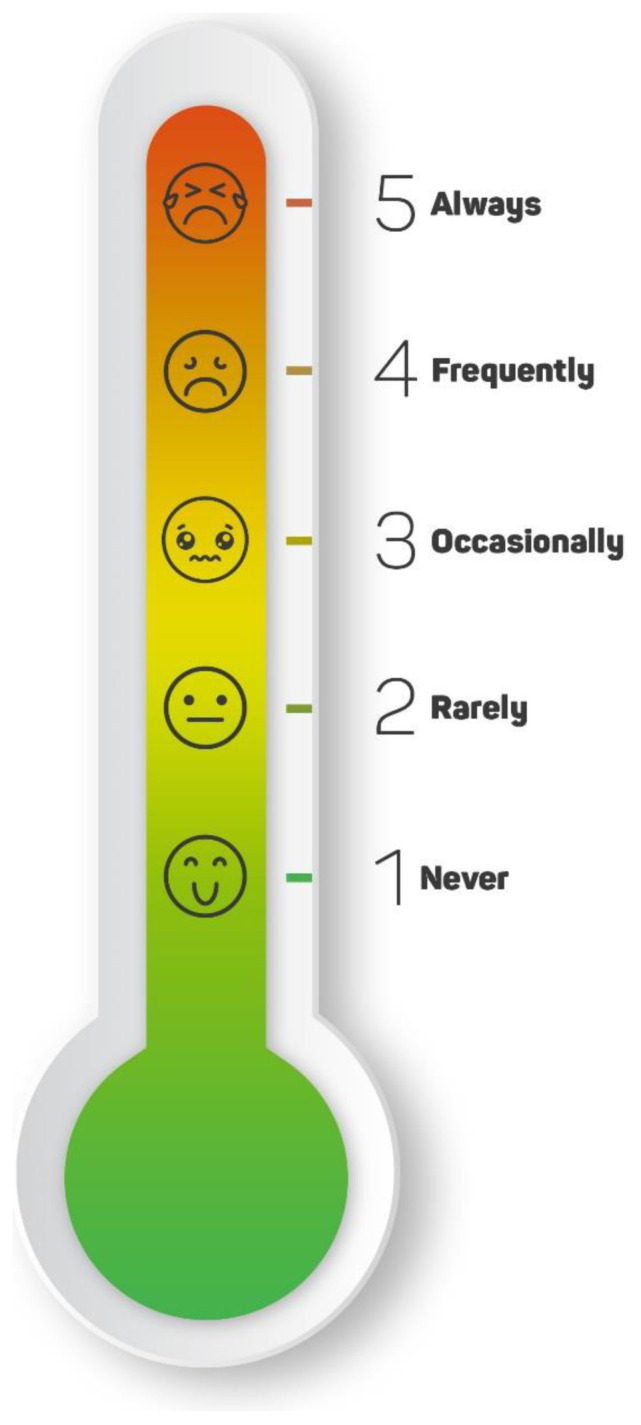
The Nausea and Vomiting Thermometer Scale

The literature was reviewed by the researcher, and general and child-specific scales related to nausea and vomiting were obtained. As a result of the literature review, a visual scale was created to measure nausea and vomiting. The scale is considered to be applicable and important for the suitability of clinical use [8,15–17]. The scale is in the form of a thermometer and has five ratings. An art director supported the design of the scale. It is scored as follows: never (1), rarely (2), occasionally (3), often (4), and always (5). In addition, as the scale score increases, the facial expressions on the scale changes. It shows: a smiley face (1), a sad face (2), an unresponsive face (3), a nauseous face (4), and a vomiting face (5). As the level of nausea and vomiting increases, facial expression becomes unhappy on the scale. For example, in the facial expression showing the 5th level, the child expresses his unhappiness by crying. The lowest score is 1 and the highest is 5. An increase in the score indicates an increase in the degree of nausea and vomiting experienced by children with cancer (Figure 1).

#### 2.3.2. Stage for forming item pool

According to Şimşek (2007), a detailed review should be carried out on the variable that will be calculated during the creation of the statements of the scale [18]. While forming the item pool of the scale, we found studies defining the nausea and vomiting scales in children with cancer. As a result of our literature review, we formed dimensions to determine the nausea and vomiting and developed item pools for use with these dimensions [8,14–17].

#### 2.3.3. Stage for Forming Specialist Opinions

At least 10 specialists recommended using a scale in order to determine the content validity of the scales [18]. We received the opinions of 11 specialists on the scales (7 academic members from the Department of Pediatric Nursing, 3 the Department of Oncology Nursing members, and 1 the Department of Pediatrics member). Specialists were given the scale type and asked to rate them between 1 and 4 to determine the convenience of the scale products (1 = requires a big shift, 4 = very convenient). The final form of the scale is as a result of the input from the specialists. The scores of the 11 experts were analyzed with the validity review of material. Expert opinions were evaluated by taking Davis technique into consideration. Davis technique grades expert opinions as (a) appropriate, (b) item should be slightly reviewed, (c) item should be seriously reviewed, and (d) item not suitable. In this technique, the number of experts marking (a) and (b) options is divided by the total number of experts, and the content validity index for the item is obtained. Instead of comparing it with a statistical criterion; a value of 0.80 is accepted as the criterion [19]. The scale-level content validity index (S-CVI) is 0.99 and it is coherent.

#### 2.3.4. Stage for forming preliminary test

Upon finding a match between the expert opinions, the scale was piloted to 30 students. Since the scale was not a concern with comprehensibility, it was considered appropriate for wide-group management. After applying the scale to a large group, the validity and accuracy test was performed. In addition, the scale was applied separately by two researchers at the same time [20].

### 2.4. Statistical Analysis

Data analysis was carried out using the IBM SPSS Statistics 21.0 (Chicago, IL) package. The percentage and mean scores for the descriptive statistics were used in the data analysis. The error margin was set to p = 0.05 when analyzing the data.

#### 2.4.1. Validity

The content validity of the scale was evaluated by experts, and the S-CVI was used in evaluating the expert opinions. The t-test was used to compare the mean scores of children with and without nausea and vomiting. The ROC analysis, the Diagnostic index (DI) and the Youden index (YI) were used for determining the scale of the cutting point. The regression analysis was used for determining the criterion or predictive validity [21].

#### 2.4.2. Reliability

We used an intra-class correlation coefficient (ICC) for determining the scale internal consistency [21]. The Bland–Altman analysis was used to evaluate the difference between the measurements taken by two different evaluators.

### 2.5. Ethics Approval

The approval of the Ethics Committee of Non-Interventional Research was obtained at the outset. In order to carry out the study, institutional permits were required. We also obtained written and verbal consent from the children and their parents by visiting them and reminding them of the study’s aims. The study was conducted in compliance with the Helsinki Declaration because the use of human beings in research includes the preservation of individual rights.

## 3. Results

### 3.1. Sample Characteristics

The sociodemographic characteristics of the children with cancer who participated in the study are given in Table 1.

### 3.2. Validity Analyses

#### 3.2.1. Content Validity

As a result of the specialists’ feedback on the draft scale, 11 expert opinions were obtained on the draft scale. The scores of the 11 specialists were assessed by content validity analysis: the S-CVI was 0.99, which was coherent.

#### 3.2.2. Cut-off point, Sensitivity, and Specificity

**Figure 2:**
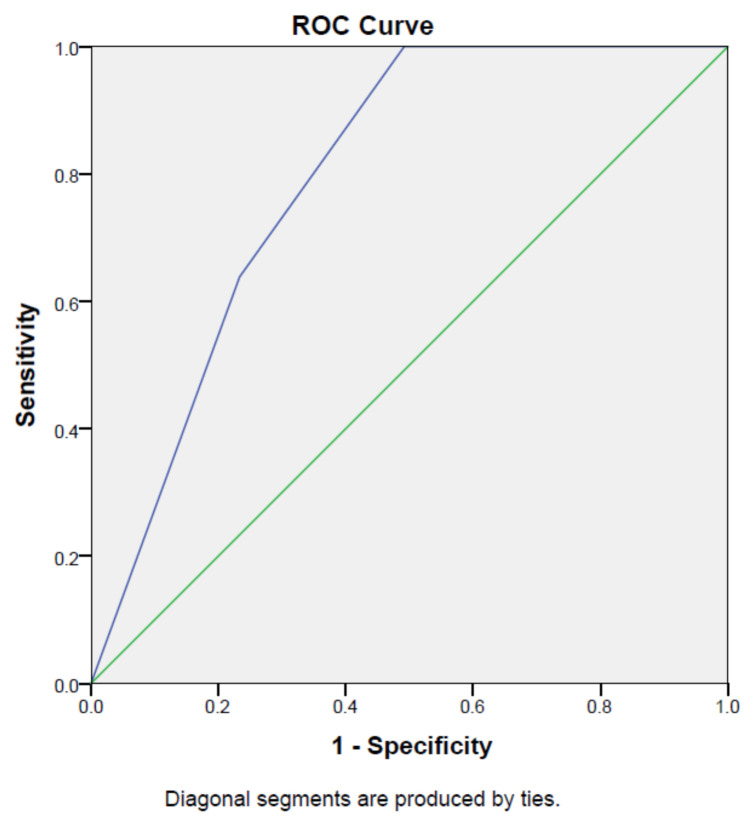
Determination of the cut-off point according to the ROC analysis.

Table 1 shows the DI and YI values determined to determine the cut-off point as a result of the ROC analysis. As the cut-off point, where the scale had the highest DI and YI values, we determined 2.5 points. We measured the sensitivity of the scale as 1.000 and the specificity of the scale as 0.942 at this point (Table 2, Figure 2). In order to reveal the real situation, children with and without nausea and vomiting were determined in the clinic. For ROC analysis, children with and without nausea and vomiting in the clinic were compared. The determined cut-off point was 3 points because 2.5 points could not be used in practice. Nausea and vomiting were found to be high in children with cancer who scored 3 points or more on the NVTS.

#### 3.2.3. Predictive Validity

In the linear regression analysis, a model was created according to the relationship between the variables. In the model, the chemotherapy drugs, nausea and vomiting type, vomiting status, and the number of children with cancer vomiting explained 44.9% of their nausea and vomiting status. It was determined that children’s chemotherapy drugs, nausea and vomiting type, vomiting status, and the number of vomiting children increased the children’s nausea and vomiting status by as much as 0.777 (β = 0.777), 0.289 (β = 0.289), 1.609 (β = 1.609), and 0.331 (β = 0.331) times, respectively. It was found that all the factors had a significant effect on nausea and vomiting status (p < 0.05, Table 3).

#### 3.2.4. Known-Group Comparison

For the known group comparison, the children were asked whether nausea and vomiting were present at the time of filling in the scale. The analysis was performed by coding the children who stated that they had nausea and vomiting as “1” and those who stated that they did not have nausea and vomiting as “0”. As a result of the analysis, it was found that there was a statistically significant difference between the mean scores of the children with ‘nausea-vomiting‘ and ‘no nausea-vomiting’ for the NVTS in children with cancer (t=10.412; p<0 .001) (Table 4).

### 3.3. Reliability Analyses

**Figure 3:**
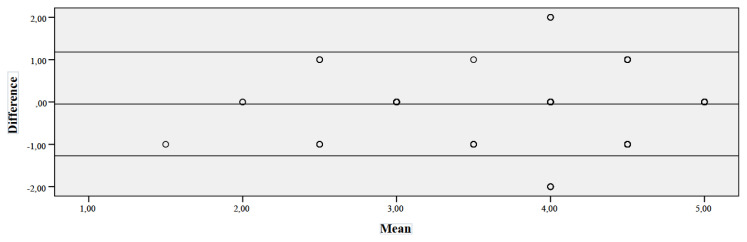
Bland-Altman plot: Difference between the NVTS’s two evaluators.

The ICC between scale was 0.99 (95% CI 0.987 to 0.992) (Table 5). As a result of the Bland–Altman analysis, the graph of the differences between the original results of the two evaluators and the results of the analysis of the differences are given in Figure 2 and Table 2. Figure 2 shows that the differences show a homogeneous distribution around zero and there is not a statistical relationship between the differences and means. As a result of the Bland–Altman analysis of the differences, it was found that the correlation coefficient between the differences and the means was insignificant (p = 0.226, Table 6). This finding supports Figure 3.

## 4. Discussion

Eleven experts assessed the material validity of the scale, and the S-CVI was used to determine the views of the experts. The S-CVI should be above 0.80 in order to suggest agreement between the experts [22,23]. In this study, the S-CVI levels were found to be above 0.80. The S-CVI results showed an agreement between the experts, the scale accurately assessed the subject, and the validity of the content was assured. According to the analysis, the expert scores were coherent. The scale is appropriate for the Turkish culture.

As a result of the ROC analysis carried out to assess the cut-off point, we defined 3 points as where the sensitivity was the highest, and the specificity was the lowest in the scale. Children with cancer, who had a score of 3 or more compared to that of the NVTS, were evaluated as having a high nausea and vomiting level.

The ROC curve provided a consistent cut-off point for the instrument evaluation, and the decisions made according to this cut-off allowed us to achieve sensitivity and specificity values. Sensitivity is described as the “condition in which those who are actually sick are also sick on the basis of the cut-off point taken during the test.” Specificity is defined as “the condition in which, as a result of the test, healthy people are also found to be healthy.” [24–26]. The curve moves upwards (high sensitivity area) and to the left (low false positivity area) as the test improves [24–26]. If the ROC curve (AUC) area is acceptable if the AUC is between 0.70 and 0.80. It is very good if the AUC is between .80 and 0.90. If the AUC is above 0.90, it is excellent [24–26]. It also had the ability to significantly distinguish the children with and without high nausea and vomiting levels.

In the literature, it is stated that factors, such as the type of chemotherapy drug, the chemotherapy protocol, the type of nausea-vomiting, the vomiting status, and the number affect the level of nausea and vomiting in children with cancer [4,5,27,28]. The logistic regression analysis conducted by Roscoe et al. indicated that expecting nausea was the strongest predictor (χ 2 = 13.15, p < 0.001) of actually developing nausea [29]. Our study shows that the NVTS was effective in the detection of nausea and vomiting levels for children with cancer. Thus, the NVTS proved to be a reliable and valid tool for determining nausea and vomiting.

In this analysis, we expected a significant difference between the mean nausea and vomiting of children with and without nausea and vomiting. This study determined the nausea and vomiting of children according to the scale cut-off point. The presence of the difference not only indicated that the scale could significantly determine the nausea and vomiting of children but also revealed the construct validity of the scale [18,20,30,31].

An ICC was used to determine the reliability of the instrument. This indicates the consistency or invariance of the measurements that an ICC obtains from individuals at the same or different times. A good fit is considered to be an ICC above 0.60. In this study, the scale were highly reliable. The ICC values indicated that the scale measured the subject sufficiently, the scale was relevant to the subject, and the scale had quite good reliability [23,32]. Therefore, the scale in this study is similar to its original construct and has a strong internal consistency.

As a result of the Bland-Altman analysis of the differences, it was found that the correlation coefficient between the differences and the means was insignificant. The Bland-Altman analysis is a scatter plot where the difference values between the measurements taken by two different methods are drawn against the average values of the same measurements. The difference between the two measurements and the random distribution of the differences around “0” provide a generalization of the research results to the whole group [33,34]. In this study, it was found that there was no relationship between the difference values and means of the scale, and it could be generalized to all children with cancer.

### 4.1. Limitations

Despite this study’s many strengths, it is limited by using random sampling, which can affect a study’s generalizability.

## 5. Conclusion

The present study revealed that the NVTS is a valid and reliable instrument to assess the level of vomiting and nausea experienced by children with cancer. It is thought to make an important contribution to pediatric oncology nurses for effective symptom management, through the creation of a visual scale that evaluates nausea and vomiting. The NVTS in children with cancer was found to be a valid and reliable measurement tool for the Turkish sample. This scale, used in pediatric oncology and hematology clinics in Turkey, will help nurses to determine nausea and vomiting in children receiving chemotherapy, and will allow them to create a common language. It is also expected to be used comfortably by pediatric oncology nurses as it is a visual scale. The use of a visual scale will allow rapid and effective evaluation of nausea and vomiting. NVTS, which is a visual analog tool, is thought to be a valuable tool, especially for studies investigating the symptoms of nausea and vomiting, since it has a very fast and easy application opportunity.

## Figures and Tables

**Table 1:** Socio-demographic characteristics of children.

		Mean	Standard Deviation
**Age**		10.68	2.60
**Day of Chemotherapy**		1.66	0.69
		**n**	**%**
**Sex**	Girls	147	58.8
	Boys	103	41.2
**Diagnosis**	Leukemias	90	36.0
	Lymphomas	24	9.6
	Solid tumors	20	8.0
	Brain tumors	36	14.4
	Soft tissue tumors	22	8.8
	Bone tumors	58	23.2
**Chemotherapy Drugs Used** *(A patient is using more than one medication)*	Cisplatin	71	28.4
	Methotrexate	62	24.0
	Ifosfamide-Cyclophosphamide	25	10.0
	Vincristine-Vinblastine	21	8.4
	Cytarabine	26	10.4
	Carboplatin	24	9.6
	Etoposide	20	8.0
	Other:	11	4.4
**Nausea-Vomiting Type**	Acute nausea-vomiting	180	72
	Delayed nausea-vomiting	68	27.2
	Anticipatory nausea-vomiting	2	0.8
**Vomiting Status**	Yes	120	48.0
	No	130	52.0
**Number of Vomiting** *(n: 120)*	0–2 times	220	88.0
	3–5 times	30	12.0

**Table 2:** The cut-off point, the estimation values, and the area under the curve (AUC) values for the prediction of nausea and vomiting according to the ROC analysis.

	Cut-off point	Sensitivity	Specificity	P	AUC* (%95 CI**)	Diagnostic Index	Youden’s Index
**NVTS**	2.5	1.00	0.942	0.000	0.794 (0.737–0.852)	1.058	0.058

* Area under curve

** Confidence Interval

**Table 3:** The extent to which children with cancer NVTS and the variables.

	NVTS				
	Model 1				
	β	Coefficients Std. Error	Standardized Coefficients Beta	t	p
**Chemotherapy Drugs**	0.777	0.015	0.252	5.225	0.000
**Nausea and Vomiting Type**	0.289	0.095	0.156	3.041	0.000
**Vomiting Status**	1.609	0.160	0.920	10.078	0.000
**Number of Vomiting Children**	0.331	0.70	0.436	4.718	0.000
**R**	**0.670**				
**R** **^2^**	**0.449**				
**F**	**49.923**				
**p**	**0.000**				
DW* **(1.5–2.5)**	**2.100**				

* Durbin Watson

**Table 4:** Comparison of the average of the high- and low-risk groups according to the determined cut-off point.

Nausea and Vomiting Status	Means			
	X	SD	t	p
**No**	3.65	0.92	10.412	0.000
**Yes**	4.63	0.48		

**Table 5:** Results of the reliability analyses of the scale.

	Intraclass Correlation	95 % CI*		F		p
		Lower Bound	Upper Bound	Value	df	
**Single measures**	0.979	0.974	0.984	95.329	249	0.000
**Average measures**	0.990	0.987	0.992	95.329	249	0.000

* Confidence Interval

**Table 6:** The results of the analysis of the differences between the original data of the two evaluators.

	Mean Difference	df	95% CI* of the Difference		t	p
			Lower	Upper		
**Difference**	− .048	249	− .1259	.0299	− 1.213	.226

* Confidence interval.
